# Training or Battling a Monster of a Location-Based Augmented-Reality Game While Descending Stairs: An Observational Study of Inattentional Blindness and Deafness and Risk-Taking Inclinations

**DOI:** 10.3389/fpsyg.2019.00623

**Published:** 2019-03-22

**Authors:** Hon-Ping Ma, Ping-Ling Chen, Václav Linkov, Chih-Wei Pai

**Affiliations:** ^1^Graduate Institute of Injury Prevention and Control, College of Public Health, Taipei Medical University, Taipei, Taiwan; ^2^Department of Emergency Medicine, Shuang-Ho Hospital, Taipei Medical University, Taipei, Taiwan; ^3^Department of Emergency Medicine, School of Medicine, Taipei Medical University, Taipei, Taiwan; ^4^Department of Traffic Psychology, CDV – Transport Research Centre, Brno, Czechia

**Keywords:** location-based augmented-reality game, inattentional blindness, inattentional deafness, risk-taking inclinations, stair walking

## Abstract

Several emerging smartphone location-based augmented-reality (AR) games require three primary tasks: training or battling a monster, capturing a monster, and searching for a monster, which involve different levels of perceptual load. Using the AR game originated from Japan as a single case study, this study examined inattentional blindness and deafness and risk-taking inclinations among participants concurrently descending stairs and engaging in these three tasks. Participants descending stairs in Taipei Medical University were observed through recordings obtained from Wi-Fi cameras to determine whether they engaged in risk-taking behaviors such as hopping, not using the handrail, and stopping suddenly. After the participants descended the stairs, they were interviewed to obtain additional information regarding demographics, game tasks (training or battling a monster, capturing a monster, or searching for a monster), data plan, and screen size. Inattentional blindness and deafness were investigated by determining whether participants saw something unusual, a police ascending the stairs, and heard the national anthem played by the police, respectively. In total, 1036 participants descended the stairs and underwent the interview between August 2016 and July 2018. Logistic regression models revealed that training or battling a monster was most associated with inattentional blindness, deafness, not using the handrail, and stopping suddenly, whereas hopping behavior was the commonest among those capturing a monster. Other contributory factors include a large smartphone screen (≥5 in), unlimited mobile data, being an undergraduate student, and an increase in the daily gaming hours. Development of smartphone apps toward detection of stair locomotion may be beneficial for curbing phone use in general and AR game playing in particular.

## Introduction

Stairways are a common place for falls; falls occur especially while descending stairs and are often associated with deaths or severe injuries (Jacobs, 2016). Studies have suggested that 41% of falls on stairs coincide with risk-taking behaviors such as hopping or not using the handrail, and phone use was one of the distractions that may potentially impair visual awareness and place additional demands on resource-limited cognitive processes (Jacobs, 2016; Lester et al., 2016). Studies examining the association between phone use, risk-taking behavior, reduced visual, and cognitive capacity during stair walking have focused on texting. For example, Hashish et al. (2017) reported that texting during stair locomotion caused changes in gait kinematics that may lead to gait disruptions, falls, and injury. Ioannidou et al. (2017) demonstrated that texting was associated with an increase in the time required to walk the stairs and a reduced use of the handrail. Lester et al. (2016) concluded that texting resulted in a significant reduction in stairway eye fixation. Haga and his colleagues (Haga et al., 2016; Haga and Matsuyama, 2018) concluded that in a laboratory setting, the number of missed visual or auditory targets was significantly greater among those texting or using Twitter during stair or treadmill walking.

While the literature has suggested that a common source of potential distraction during stair locomotion was texting, recently introduced augmented-reality (AR) smartphone games may reduce visual attention and cognitive capacity to a greater degree. Using geolocation, the pioneering AR game originated from Japan creates augmented-reality gaming scenarios in which players are required to engage in several game tasks. For example, players have to walk designated distances to hatch eggs; players place incense or lure module to search for a monster; players launch a ball to capture a free-roaming monster; and to conquer or defend a gym, players have to train their monster and fight others’ monster. Behavioral studies examining the effects of this particular game have been specific to pedestrians crossing signalized intersections (Chen and Pai, 2018; Chen et al., 2018) and uncontrolled intersections (Chen and Pai, 2017). These studies have reported that playing this particular smartphone AR game may lead to several risky street-crossing behaviors, such as not using the designated crossing or crossing on red at a signalized intersection (Chen and Pai, 2018; Chen et al., 2018), exhibiting few head-turning frequencies, not looking at traffic before crossing, and failing to look at the correct direction of traffic at an uncontrolled intersection (Chen and Pai, 2017).

In a series of neuroscience studies by Macdonald and Lavie (2011), Lavie et al. (2014), Molloy et al. (2015), Raveh and Lavie (2015), a low- or high-load visual discrimination condition involving a cross shape (with varying line colors or lengths) was presented to the participants to examine inattentional deafness. A significantly higher rate of participants in the high-visual-load conditions, compared with those in the low-visual-load conditions, failed to notice the presence of a brief and pure tone, a phenomenon of inattentional deafness. In a series of behavioral studies examining inattentional blindness, Hyman et al. (2014a,b) reported that during level walking, those talking or texting on a phone were less likely to notice an unusual object (a clown on a unicycle or money in a tree), compared with individuals engaging in other tasks such as listening to music. The above-mentioned studies (Macdonald and Lavie, 2011; Hyman et al., 2014a,b; Molloy et al., 2015; Raveh and Lavie, 2015) have consistently suggested that high perceptual load in tasks may consume all or most of the inattentional capacity, thereby resulting in reduced perception of task-irrelevant visual stimuli (inattentional blindness) and auditory stimuli (inattentional deafness).

We adopt the experiment design from Lavie et al. (2014) by assuming that the three distinct game tasks have different levels of interactivity (i.e., different levels of complexity) with players. It is possible that training or battling a monster is the most complicated task that requires the highest cognitive-perceptual demand because the task involves extensive tapping of the screen and swiping fingers to the left or right to avoid attack from a rival. Other two tasks, i.e., capturing a monster or searching for a monster, would not require as high perceptual load as the task of battling a monster. The task of capturing a monster requires players to throw a ball toward a monster, but players do not have to dodge attacks from the monster, which is a routine feature in a battling mode. Searching for a monster would require the least cognitive-perceptual demand because players just have to place incense or launch lure module. Our primary research hypothesis is that training or battling a monster is the task most associated with inattentional blindness, deafness, and risk-taking behaviors such as not using the handrail or changes in gait kinematics (hopping or stopping suddenly). To ascertain this research hypothesis, this study examined inattentional blindness and deafness and risk-taking behaviors among players who were concurrently descending stairs and engaging in several game tasks.

### Purpose

In an extension to the work by Chen and Pai (2018), the current study investigated the effects of three primary and distinct tasks of the particular AR game (training/battling a monster, capturing a monster, and searching for a monster) on inattentional blindness and deafness and several risk-taking inclinations while descending stairs. We focused on participants descending stairs because epidemiological studies (e.g., Blazewick et al., 2018) have reported that falls from stairs are more prevalent when walking down the stairs compared with when walking up the stairs.

## Materials and Methods

### Participants

Following the data-collection method of our previous work (Chen and Pai, 2018), we observed and surveyed participants seen descending stairs within the main campus of Taipei Medical University, Taiwan, where monsters and gyms of this particular game commonly appear for players to catch and battle. This method involved both observation and a face-to-face interview/survey of participants observed to be using their smartphone while descending stairs. We describe the data-collection process in detail below.

To collect data on participants’ risk-taking behaviors (including hopping, not using the handrail, and stopping suddenly) when descending stairs, three camera devices (relevant model: D-Link DCS-2630L Full HD 180-Degree Wi-Fi Camera) were installed along the stairway to record videos. These three camera devices were installed at the start, middle, and end points, respectively. Participants descending stairs were classified into two groups: the undistracted group (that serves as the control group) consisting of those who were just descending stairs and not using their smartphones at all; and the distracted group (the case group) consisting of those using their smartphones. To avoid selection bias, participants were selected at random by using an online random number generator.

A participant who had finished descending the stairs was approached by the interviewers and was invited for a face-to-face interview. The interviewers were informed by the observers in the laboratory (who watched the real-time video clips and performed the random sampling) regarding which participants to invite for an interview. If multiple participants were observed using their smartphones, only one was randomly selected for the interview. The participants were interviewed and asked what they were just doing with their smartphones. Those who confirmed to be playing the game were then asked to indicate what game tasks (training/battling a monster, capturing a monster, and searching for a monster) they were engaging in. Participants were also asked to report other information such as smartphone features (e.g., screen size, data plan, duration of gaming daily, and demographic data).

To evaluate participants’ inattentional blindness, we recruited a male research assistant, with a height of 170 cm wearing a police outfit and walking up the stairs while the participant was descending the stairs. To examine participants’ inattentional deafness, the same police played the Taiwan national anthem from his smartphone at approximately 60 dBA and at a distance of 1 m. The presence of the police and national anthem served as an unusual visual and auditory stimulus to evaluate participants’ inattentional blindness and deafness, respectively. Participants who completed descending the stairs were interviewed to determine whether they had seen the police and heard the national anthem. Participants received a pen (price: ∼US$1) as a compensation for their interview time. Regarding consent to participate, the interviewers explained the study to the participants in greater detail as soon as they were invited for the interview. Participants were then asked to sign a consent form acknowledging their understanding of the research as well as the questionnaire. All participants consented to participate in the study, provided that no personal information, images, or video footage were revealed. Written consent was obtained from each participant. The study was approved in its entirety by the Institutional Review Board affiliated with Taipei Medical University (IRB#:n201510012).

**Figure 1:**
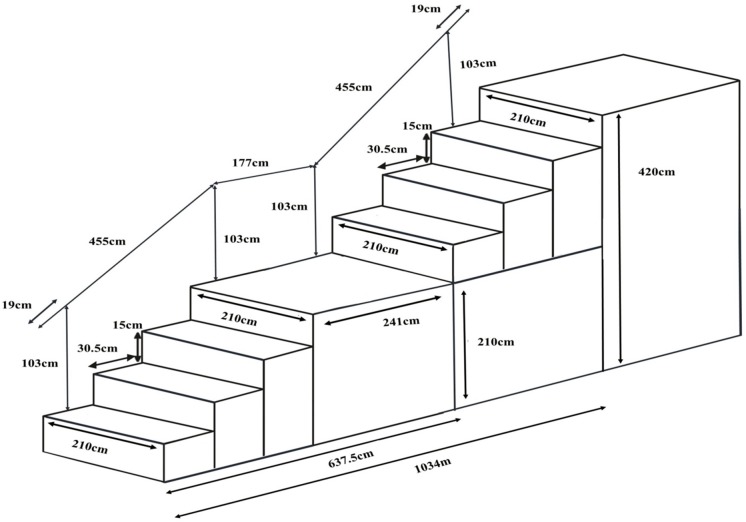
Designated stairway where participants were observed and interviewed.

We collected the data from August 2016 to July 2018. Two stairways were approved by the university for our Data Collection. However, because of budget and manpower limitations, only one stairway was randomly chosen. The half landing stairway, which connects the university library on the first floor with the ground floor, has the following dimensions (Figure 1): length, height, and width of approximately 10.34, 4.20, and 2.10 m, respectively, 26 steps (a rise of 15 cm and a run of 30.5 cm); a slope of approximately 26°; and handrails with 1.03-m height on both sides. The stair landing is 2.10 m wide and 2.41 m long. For brevity, Figure 1 illustrates three steps for each of the two stairway parts.

**Figure 2:**
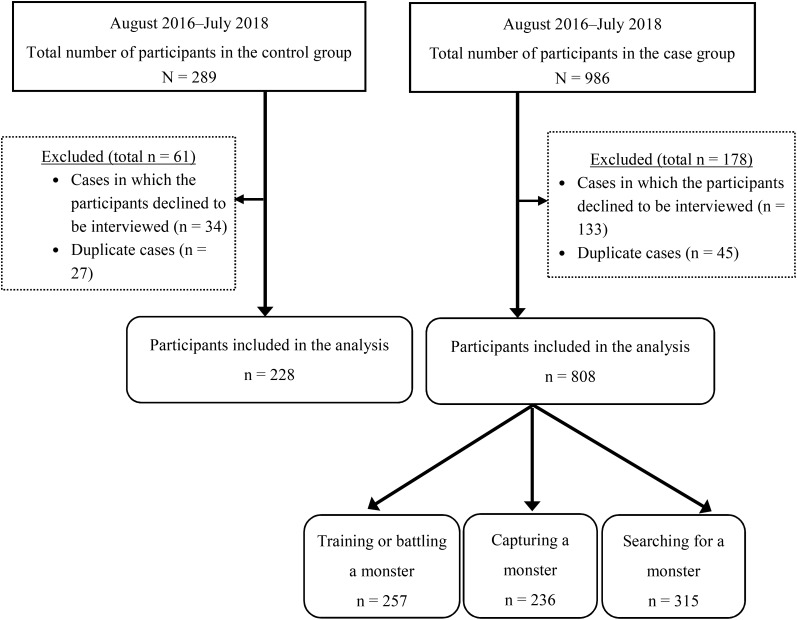
Study flow chart.

Figure 2 shows the flow-chart of our sample selection. In the case group (game players), 986 participants were initially included and confirmed to be playing the game. In total, 133 participants declined to be interviewed further for additional variables and were thus excluded from this study. We further excluded 45 participants who were previously observed and interviewed. A total of 808 valid participants remained in the case group. Of the 808 participants, 257 were training or battling a monster, 236 were capturing a monster, and 315 were searching for a monster. In the control group, 289 participants were enrolled. Those who declined to be interviewed (*n* = 34) and those who had been previously observed and interviewed (*n* = 27) were excluded. This yielded 228 participants in the control group. In total, 1036 valid participants descended the stairs and underwent the interview.

### Variables Considered

The measured independent variables included gender, age, student status, month data allowance, smartphone screen size, game tasks, and daily duration of playing the game. University academic or administration staff who reported to be playing the game was relatively rare and were therefore excluded. Only students were considered in this study. Students were classified into two levels: undergraduate and postgraduate. Two continuous variables were considered, namely age (y) and daily duration of playing the game (hour). As a categorical variable, screen size (diagonal length) was classified as < 5 inches and ≥ 5 inches. Mobile data allowance was classified as restricted or unlimited. Data plan is the variable of interest in the current research, primarily because a recent study (Gariazzo et al., 2018) has reported a strong relationship between Internet volume and road crash fatalities. It is possible that different data plans are associated with inattentional blindness/deafness and risk-taking behaviors while descending stairs.

This study examined three distinct game tasks, namely training or battling a monster, capturing a monster, and searching for a monster. Training or battling a monster was defined as training their own monster or battling other monster. Capturing a monster was defined as heading toward the location for capturing a monster or attempting to catch a monster using a dedicated ball. Searching for a monster was defined as searching for a monster by using incense or placing lure module. We excluded the task “hatching an egg” because this can be done concurrently with other tasks.

Two sets of outcome measures, namely risk-taking behaviors and inattentional blindness/deafness, were examined. Risk-taking behaviors consisted of hopping, not using the handrails, and stopping suddenly. The hopping behavior was defined as descending the stairs not one tread at a time. The behavior of not using the handrail was defined not using the handrail at all. The behavior of stopping suddenly was defined as suddenly stopping at any point while descending the stairs. Those who stopped suddenly due to being blocked by others were not considered as stopping suddenly. Participants who reported to have not seen the police at all were classified as those who sustained inattentional blindness, and those who reported not having heard the Taiwan national anthem were classified as those who sustained inattentional deafness.

### Analysis

The distribution of various game tasks using other independent variables was first reported. The percentages of the outcome variables according to the case (those engaging in the three game tasks) and control (undistracted participants) groups were then evaluated. Chi-square test was adopted *post hoc* to determine significant differences among game tasks and outcome variables. Subsequently, we estimated logistic regressions to examine factors predicting the outcome variables: risk-taking behaviors and inattentional blindness and deafness.

Univariate logistic regressions were estimated to identify the contributory factors to the outcome variables. Any variable with *p* < 0.2 was incorporated into the multivariate logistic analysis, and significant variables with *p* < 0.1 were retained for the final regression analysis. For conciseness, univariate regression results are not presented.

## Results

### General Results

Table 1 lists the distribution of game tasks according to the other independent variables. Of the participants, 24.81, 22.78, and 30.41% claimed to be training or battling a monster, capturing a monster, and searching for a monster, respectively. Of those playing the game, 71.75% had unlimited data allowance, which is the highest compared with all the other tasks.

**Table 1:** Distribution of various types of game tasks according to independent variables (*N* = 1036).

Characteristics		Control (%)	Game tasks			Total (%)
			Training/battling a monster (%)	Capturing a monster (%)	Searching for a monster (%)	
Gender	Male	131 (57.46)	145 (56.42)	134 (56.78)	174 (55.24)	584 (56.37)
	Female	97 (42.54)	112 (43.58)	102 (43.22)	141 (44.76)	452 (43.63)
Age (y)^a^		m: 23.1; s: 1.3	m: 20.4; s: 0.9	m: 20.6; s: 1.3	m: 21.2; s: 2.1	m: 21.2; s: 2.4
Student status	Undergraduate	135 (59.21)	155 (60.31)	139 (58.90)	188 (59.68)	617 (59.56)
	Postgraduate	93 (40.79)	102 (39.69)	97 (41.10)	127 (40.32)	419 (40.44)
Day of the week	Weekdays	162 (71.05)	161 (62.65)	153 (64.83)	210 (66.67)	686 (66.22)
	Weekends	66 (28.95)	96 (37.35)	83 (35.17)	105 (33.33)	350 (33.78)
Screen size	< 5 inches	78 (34.21)	91 (35.41)	73 (30.93)	99 (31.43)	341 (32.92)
	≥ 5 inches	150 (65.79)	166 (64.59)	163 (69.07)	216 (68.57)	695 (67.08)
4G Data allowance	Unlimited use	149 (65.35)	170 (66.15)	152 (64.41)	226 (71.75)	697 (67.28)
	Restricted use	79 (34.65)	87 (33.85)	84 (35.59)	89 (28.25)	339 (32.72)

Time spent on the game daily (h)^a^		m: 0.4; s: 0.3	m: 5.4; s: 2.3	m: 4.8; s: 2.3	m: 5.8; s: 4.7	m: 4.9; s: 3.6

Total (%)		228 (22.01)	257 (24.81)	236 (22.78)	315 (30.41)	1036 (100)

^a^*The figures represent the mean (m) and standard deviation (s)*.

To examine participants’ risk-taking behaviors and inattentional blindness and deafness, the proportions of these outcome variables by various game tasks are presented in Table 2. In the “hopping” category, 72.0% for capturing a monster means that 72.0% of the participants who were capturing a monster had hopping behavior when descending the stairs, which was the highest among all thee game tasks. Participants training or battling a monster were most likely to not use the handrail (92.6%, *p* < 0.01) and to stop suddenly (76.7%, *p* < 0.01). Inattentional blindness (not seeing the police) and deafness (not hearing the song) were exhibited by 78.2 and 73.5% of those training or battling a monster (*p* < 0.01), followed by 40.9 and 41.9% of those capturing a monster (*p* < 0.01), respectively.

**Table 2:** Risk-taking behaviors and inattentional blindness/deafness of the case and control groups.

	Hopping (%) (*n* = 358)	Not using the handrail (%) (*n* = 761)	Stop suddenly (%) (*n* = 364)	Inattentional blindness (%) (*n* = 398)	Inattentional deafness (%) (*n* = 405)
Control	13.6	42.5	10.1	4.4	7.5
Case					
Training/battling a monster	18.3	92.6**	76.7**	78.2**	73.5**
Capturing a monster	72.0**	83.9**	13.4	40.9**	41.9**
Searching for a monster	35.2**	72.7**	35.9**	28.6*	31.4*

^∗^*p < 0.05 in relation to the control group. ^∗∗^*p* < 0.01 in relation to the control group*.

### Model Estimation Results for Risk-Taking Behaviors

Table 3 reports the odds of risk-taking behaviors by using multiple logistic models. Capturing a monster was most likely to lead to hopping behaviors (adjusted odds ratio [AOR] = 4.36, confidence interval [CI] = 1.98–9.61). Participants who were training or battling a monster most frequently exhibited behaviors of not using the handrail (AOR = 2.84, CI = 1.52–5.30) and stopping suddenly (AOR = 6.82, CI = 2.32–20.05).

**Table 3:** Odds of the three risk-taking behaviors.

	Hopping		Not using the handrail		Stopping suddenly	
Game tasks (ref.: control)	AOR (95% CI)	*p* value	AOR (95% CI)	*p* value	AOR (95% CI)	*p* value
Training/battling a monster	1.11 (0.96, 1.28)	0.17	2.84 (1.52, 5.30)	<0.01	6.82 (2.32, 20.05)	<0.01
Capturing a monster	4.36 (1.98, 9.61)	<0.01	2.55 (1.39, 4.68)	<0.01	1.05 (0.96, 1.14)	0.26
Searching for a monster	2.98 (1.51, 5.87)	<0.01	2.05 (1.28, 3.28)	<0.01	2.35 (1.29, 4.27)	<0.01
Undergraduate (ref.: otherwise)	2.39 (1.25, 4.55)	0.02	1.90 (1.13, 3.20)	0.03	–	–
Screen size of ≥ 5 inches (ref. otherwise)	1.78 (1.14, 2.78)	0.01	1.60 (1.01, 2.52)	0.04	1.33 (1.25, 1.80)	0.02
Screen size of ≥ 5 inches × capturing a monster (ref.: otherwise)	3.96 (1.69, 9.30)	<0.01	2.79 (1.47, 5.28)	<0.01	–	–
Screen size of ≥ 5 inches × training/battling a monster (ref.: otherwise)	–	–	3.33 (1.50, 7.41)	<0.01	5.58 (1.98, 15.69)	<0.01
Unlimited data allowance (ref.: limited)	2.32 (1.27, 4.23)	<0.01	2.54 (1.32, 4.89)	<0.01	–	–
Unlimited data × capturing a monster (ref.: otherwise)	2.65 (1.44, 4.89)	<0.01	–	–		
Unlimited data × training/battling a monster (ref.: otherwise)	–	–	2.15 (1.31, 3.53)	<0.01	–	–
Time spent on the game daily	1.09 (1.03, 1.16)	<0.01	1.05 (1.01, 1.10)	0.03	–	–
ρ^2^	0.268		0.331		0.214	

Compared with postgraduates, undergraduate students exhibited higher odds of hopping (AOR = 2.39, CI = 1.25–4.55) and not using the handrail (AOR = 1.90, CI = 1.13–3.20). Participants with smartphone screens of ≥ 5 inches exhibited an increased likelihood of engaging in the three risk-taking behaviors (AORs = 1.78 for hopping, 1.60 for not using the handrail, and 1.33 for stopping suddenly). The interaction term “large screen size and training/battling a monster” appears to be statistically significant: individuals training/battling a monster with their smartphone having a screen size of ≥ 5 inches exhibited an increased likelihood of not using the handrail (233%; AOR = 3.33, CI = 1.50–7.41) and stopping suddenly (458%; AOR = 5.58, CI = 1.98–15.69). In addition, large phone sizes and capturing a monster appear to be significantly associated with an increased likelihood of hopping (296%; AOR = 3.96, CI = 1.69–9.30) and not using the handrail (179%; AOR = 2.79, CI = 1.47–5.28).

Compared with those having restricted data allowance, participants having unlimited data allowance were 2.32 (AOR = 2.32, CI = 1.27–4.23) and 2.54 (AOR = 2.54, CI = 1.32–4.89) times more likely to hop and to fail to use the handrail, respectively. The interaction effect of unlimited data, capturing a monster, and training/battling a monster contributed to certain risk-taking behaviors. Participants capturing a monster and training/battling a monster with their Internet data unrestricted were 2.65 (AOR = 2.65, CI = 1.44–4.89) and 2.15 (AOR = 2.15, CI = 1.31–3.53) times more likely to hop and to fail to use the handrail, respectively.

Participants’ daily duration of playing the game contributes to hopping and not using the handrail. An increase in the hour of playing the game resulted in an increased likelihood of hopping by 9% (AOR = 1.09, CI = 1.03–1.16) and not using the handrail by 5% (AOR = 1.05, CI = 1.01–1.10).

### Model Estimation Results for Inattentional Blindness and Deafness

Table 4 reports the model estimation results for inattentional blindness and deafness. Training or battling a monster was most likely to result in failure to see the police (AOR = 16.32, CI = 3.03–87.92) and hear the song (AOR = 9.16, CI = 2.32–36.18). Capturing a monster was second most likely to lead to inattentional blindness (AOR = 7.34, CI = 1.99–27.11) and deafness (AOR = 4.45, CI = 1.69–11.73).

**Table 4:** Odds of inattentional blindness and deafness.

	Inattentional blindness		Inattentional deafness	
Game tasks (ref.: control)	AOR (95% CI)	*p* value	AOR (95% CI)	*p* value
Training/battling a monster	16.32 (3.03, 87.92)	<0.01	9.16 (2.32, 36.18)	<0.01
Capturing a monster	7.34 (1.99, 27.11)	<0.01	4.45 (1.69, 11.73)	<0.01
Searching for a monster	4.21 (1.22, 14.49)	0.02	4.09 (1.13, 14.77)	0.03
Undergraduate (ref.: otherwise)	1.88 (1.24, 2.86)	<0.01	1.46 (1.03, 2.08)	0.05
Screen size of ≥ 5 inches (ref. otherwise)	3.43 (1.39, 8.45)	0.01	2.67 (1.37, 5.21)	<0.01
Screen size of ≥ 5 inches × training/battling (ref.: otherwise)	8.67 (2.27, 33.10)	<0.01	7.72 (2.29, 25.99)	<0.01
Unlimited data allowance (ref.: limited)	2.79 (1.10, 7.08)	0.03	3.16 (1.02, 9.81)	0.05
Unlimited data × training/battling (ref.: otherwise)	5.77 (2.06, 16.39)	<0.01	6.94 (2.24, 21.49)	<0.01
Time spent on the game daily	1.19 (1.01, 1.41)	0.04	1.13 (1.04, 1.22)	<0.01
ρ^2^	0.229		0.274	

Similar to the models of risk-taking behaviors, undergraduate students exhibited an increased likelihood of inattentional blindness (AOR = 1.88, CI = 1.24–2.86) and deafness (AOR = 1.46, 1.03–2.08). Compared with participants with a screen size of < 5 inches, those with ≥ 5-in screens demonstrated a higher tendency to sustain inattentional blindness (AOR = 3.43, CI = 1.39–8.45) and deafness (AOR = 2.67, CI = 1.37–5.21). Furthermore, compared with participants engaging in training or battling a monster with < 5 inch screens, those with ≥ 5-inch screens exhibited an increased likelihood of not noticing the police (767%; AOR = 8.67, CI = 2.27–33.10) and not hearing the national song (672%; AOR = 7.72, CI = 2.29–25.99).

Participants with unlimited Internet data sustained inattentional blindness and deafness, respectively, 2.79 (AOR = 2.79, CI = 1.10–7.08) and 3.16 (AOR = 3.16, CI = 1.02–9.81) times more, respectively, than participants with restricted mobile Internet data. We observed a significant interaction between unlimited data and training/battling a monster; players possessing unlimited Internet data allowance and training/battling a monster were 5.77 (AOR = 5.77, CI = 2.06–16.39) times more likely to not to notice the police. Furthermore, this interaction term contributed to participants’ auditory inattention (AOR = 6.94, CI = 2.24–21.49). An increase in the hour of playing the game was found to lead to an increased likelihood of not noticing the police by 19% (AOR = 1.19, CI = 1.01–1.41) and not hearing the national song by 13% (AOR = 1.14, CI = 1.04–1.22).

## Discussion

The literature has demonstrated that visual and auditory detection sensitivity was reduced with high visual perceptual load (Raveh and Lavie, 2015). Our primary research hypothesis is that training or battling a monster is the task most associated with inattentional blindness, deafness, and risk-taking behaviors such as not using the handrail or changes in gait kinematics (hopping or stopping suddenly). We conducted an observational study followed by an interview, establishing that participants training or battling a monster, a task of high visual perceptual load, were most likely to miss visual (a police walking up the stairs) or auditory (a national song) targets while descending the stairs. Our finding here reveals that visual and auditory detection sensitivity was consistently reduced with the game task involving the highest perceptual load. Compared with the other two game tasks (searching for a monster and capturing a monster), training or battling a monster involved the highest perceptual load because to engage in such a battling task, players launch attacks by substantially tapping on the touchscreen or dodge attacks from other monster by swiping fingers to the left or right.

Our findings reveal that training or battling a monster was the task most associated with failing to use the handrail and stopping suddenly when descending the stairs. Such findings are intuitive because battling a monster, compared with the other two game tasks, can be most manually demanding, which deters participants from using the handrail. In addition, because the task was most cognitively and manually demanding, participants compensated for the increased risk of falls by stopping suddenly. Conversely, the task of capturing a monster was most associated with the hopping behavior. Such a finding can be reasonable because participants may have to rush to locations where a monster appears but can disappear immediately. Our findings here support our primary research hypothesis that game task with high visual and manual demand may induce risk-taking behaviors such as not using a handrail or changes in gait kinematics (hopping or stopping suddenly). Previous studies (Haga et al., 2016; Hashish et al., 2017; Ioannidou et al., 2017; Haga and Matsuyama, 2018) have demonstrated that secondary-task engagement such as texting messages was associated with several risk-taking behaviors such as reduced use of handrail or eye fixation on stairs. Moreover, our findings provide evidence regarding the critical role of certain game tasks (that require high visual and manual demands such as battling or capturing a monster) in causing unsafe stair walking.

Our results are congruent with the findings of studies that have examined pedestrians’ street-crossing behaviors (Chen and Pai, 2018; Chen et al., 2018), demonstrating that specific game tasks, such as training or battling a monster and capturing a monster, were associated with inattentional blindness and deafness and several risk-taking inclinations while descending the stairs. Taking advantage of smartphone cameras, apps have been developed to remind pedestrians of potential risks of using a phone while crossing a street (Wang et al., 2012; Zhou, 2015). Future development of such a technology toward detection of stair locomotion may be beneficial for curbing phone use in general and location-based AR game playing in particular.

Other findings need further additional discussion here. For instance, we observed an association of unlimited data allowance and two tasks, i.e., battling a monster or capturing a monster, with risk-taking inclinations and inattentional blindness and deafness. Furthermore, Chen and Pai (2018) identified this combined effect as a risk factor for unsafe street-crossing behavior; therefore, attention should be given to the particular AR game players whose Internet data usage is particularly high.

We observed that larger smartphone screens (i.e., ≥5 inches) itself, as well as the interaction effect of this factor with battling a monster, increased the likelihood of risk-taking inclinations and inattentional blindness and deafness. Studies focusing on smartphone marketing have reported that because large screens facilitate both hedonic and utilitarian uses of smartphones, they are more likely than smaller screens to entice people to adopt smartphones (Kim and Sundar, 2014). We speculate that game players with larger screens have large mobile data allowances and therefore more likely to sustain inattentional blindness and deafness than are players with small screens. Future studies analyzing screen size, usage patterns, and behavior are required to ascertain our speculation.

Studies (e.g., Demirci et al., 2015) have suggested that smartphone addiction among students is associated with depression, anxiety, and sleep problems. Our study further demonstrated that game players who were undergraduate students were associated with certain risk-taking behaviors and inattentional blindness and deafness. Student players of this certain AR game, in particular undergraduate students, should be educated regarding the risk involved. In addition, our findings reveal that an increase in the hour of playing the game every day exposed participants to an increased risk of certain risk-taking behaviors and inattentional blindness/deafness while descending stairs. The literature (Gentile, 2009) suggested that university students addicted to videogames had trouble paying attention in classes and were more likely to be diagnosed as having an attention disorder. It is out of the scope of the current research to identify whether the participants were pathological AR game players. However, platform players should be aware of the uniquely immersive and addictive nature of this game.

To the best of our knowledge, this is the first study to examine load-induced inattentional blindness and deafness as well as risk-taking inclinations among participants engaging in certain game tasks while descending stairs. Our study contributes to existing knowledge by demonstrating that visual and auditory detection sensitivity was reduced with certain game tasks (e.g., battling or searching for a monster) that require high visual and manual perceptual loads. Other location-based AR games share the similar features of this particular AR game such that players have to battle a dinosaur/zombie, capture a dinosaur/zombie, or search for a dinosaur/zombie in the virtual environment. Our findings can be generalized to other location-based AR games that have similar game tasks.

The current research, however, is not without its limitations. First, we both observed participants and later interviewed them. Causal inference was not possible; therefore, we investigated simple correlations. In addition, despite adopting random sampling, not all game players and undistracted participants were selected, because it was impossible to observe all participants descending the stairs. This was another inevitable research limitation. The third limitation is that the research began in August 2016, immediately following the unprecedented growth in the popularity of this game (though after an obvious decline in user base recently). Undoubtedly, our data are representative only of the peak period (possibly the first year of the study), but we argue that if other location-based AR games reach a similar level of popularity, our data may be extended to the safety risks of playing. Furthermore, it would be crucial to examine whether AR games lead to more risks compared to non-AR games. We have addressed this comparison in our previous work (Chen and Pai, 2018) for pedestrians crossing a signalized street. Our main findings in our previous work include that compared to those playing other non-AR apps, AR game players were more likely to engage in several risk-taking behaviors. However, due to restricted research funding, it is out of scope of the current research to observe and interview participants who are concurrently playing non-AR applications and descending stairs. Past behavioral studies (e.g., Hyman et al., 2014a,b; Ioannidou et al., 2017; Haga and Matsuyama, 2018; Jiang et al., 2018; Wells et al., 2018; Feld and Plummer, 2019) have adopted video cameras to examine how secondary tasks such as texting messages affect cognitive ability and induce risk-taking behaviors. By extending these studies, we adopted the methods featuring both an observational component followed by an interview/survey of those observed to be playing the game while descending stairs. With this astute approach, our research hypothesis and conjecture can be confirmed by our findings. Nonetheless, our findings can be validated in future research that may test psychometric scales for the analysis of different levels of interactivity. Finally, our study was not able to control other crucial variables, for example, legendary monster that can be rarely seen and lead to higher levels of load-induced inattentional blindness and deafness than a common monster. We were unable to extend our study to other stairways, different dimensions of which are likely to result in variations in risk-taking behaviors and inattentional blindness/deafness. In addition, when examining participants’ auditory detection sensitivity, we were unable to control for other ambient acoustic stimuli such as noise from the crowd.

## Conclusion

In conclusion, among several game tasks, training/battling a monster was the task most associated with inattentional blindness/deafness and certain risk-taking behaviors (e.g., not using the handrail and stopping suddenly), whereas capturing a monster was the task most associated with hopping. We strongly recommend that AR game playing should not be permitted when descending stairs.

## Author Contributions

H-PM and P-LC drafted and revised the manuscript and established the theoretical supports for data analyses. VL edited the manuscript, reviewed relevant literature, and strengthened discussions and conclusions. C-WP was responsible for study design, contributed to the analyzing and interpretation of data, and drafted the manuscript. The final version of the manuscript was read and approved by each contributing author.

## Conflict of Interest Statement

The authors declare that the research was conducted in the absence of any commercial or financial relationships that could be construed as a potential conflict of interest.
